# A Systematic Review on Traumatic Brain Injury Pathophysiology and Role of Herbal Medicines in its Management

**DOI:** 10.2174/1570159X21666230126151208

**Published:** 2023-09-25

**Authors:** Kaushal Arora, Vishal Vats, Nalin Kaushik, Deepanshu Sindhawani, Vaishali Saini, Divy Mohan Arora, Yogesh Kumar, Etash Vashisht, Govind Singh, Prabhakar Kumar Verma

**Affiliations:** 1 Department of Pharmaceutical Sciences Maharshi Dayanand University, Rohtak, Haryana, 124001, India;; 2 Department of Pharmaceutical Sciences, Chaudhary Bansi Lal University, Bhiwani, Haryana, 127031, India;; 3 Department of Pharmaceutical Sciences Guru Jambheshwar University of Science & Technology, Hisar, Haryana, 125001, India;; 4 Sat Priya College of Pharmacy, Rohtak, Haryana, 124001, India

**Keywords:** Traumatic brain injury, medicinal herbal plants, neurodegenerative disorder, pathophysiology, inflammation, oxidative stress, glucose metabolism

## Abstract

**Background:**

Traumatic brain injury (TBI) is a worldwide problem. Almost about sixty-nine million people sustain TBI each year all over the world. Repetitive TBI linked with increased risk of neurodegenerative disorder such as Parkinson, Alzheimer, traumatic encephalopathy. TBI is characterized by primary and secondary injury and exerts a severe impact on cognitive, behavioral, psychological and other health problem. There were various proposed mechanism to understand complex pathophysiology of TBI but still there is a need to explore more about TBI pathophysiology. There are drugs present for the treatment of TBI in the market but there is still need of more drugs to develop for better and effective treatment of TBI, because no single drug is available which reduces the further progression of this injury.

**Objective:**

The main aim and objective of structuring this manuscript is to design, develop and gather detailed data regarding about the pathophysiology of TBI and role of medicinal plants in its treatment.

**Methods:**

This study is a systematic review conducted between January 1995 to June 2021 in which a consultation of scientific articles from indexed periodicals was carried out in Science Direct, United States National Library of Medicine (Pubmed), Google Scholar, Elsvier, Springer and Bentham.

**Results:**

A total of 54 studies were analyzed, on the basis of literature survey in the research area of TBI.

**Conclusion:**

Recent studies have shown the potential of medicinal plants and their chemical constituents against TBI therefore, this review targets the detailed information about the pathophysiology of TBI and role of medicinal plants in its treatment.

## 1 INTRODUCTION

Traumatic brain injury is the major cause of morbidity and mortality among children and adolescents, defined as a head injury due to penetration and diffuse trauma. Penetrating trauma occurs due to the insertion or laceration of sharp and blunt objects, and diffuse trauma occurs mainly due to closed head injury in which skull fracture does not occur. In brain injury, most falls occur primarily in young children and older adults, while road traffic accidents are the major reason for head injury [1].

The primary injury includes sudden injury to the irreversible brain, which may manifest as intraparenchymal contusion, diffuse axonal injury, and skull intracranial hematomas. This mechanical injury cause changes in the brain and produces secondary injury, which includes various complex cascades that affect brain physiology at different intervals, including microglia activation, the release of pro-inflammatory mediators, an increase in oxidative stress, edema, and several other factors that generate further damage [2]. Metabolic changes also occur in the brain due to alterations in the cell metabolism and disruption of metabolic energy demand. A decrease in metabolic energy demand upregulates inflammatory activity and increases oxidative damage. In a neuron, activation of microglia causes the release of pro-inflammatory cytokines in response to inflammation. Due to this, BBB is compromised, and inflammatory cells invade and change the physiology of the brain. Due to these changes, water content rises in the brain physiology [3].

In developing countries, road traffic accidents are a major cause, while in developed countries, falls and sports-related injuries are important reasons for TBI.

## 2 MATERIALS AND METHODS

For a better understanding of the pathophysiology of TBI, various animal models are used. The most commonly used animals in these models are rats and mice because of their low cost, easy availability, shorter life span, and ease of handling [4]. Fluid percussion, the weight drop method, controlled cortical impact, and the blast wave injury model are the most widely used methods to induce TBI. Every model has its own positive and negative aspects. The weight drop method induces diffuse injury, similar to injury in road traffic accidents, and is a non-penetrating model. On the other hand, fluid percussion and controlled cortical impact model induce focal injury, which is clinically relevant to sports-related injury [5]. The blastwave injury model also causes diffuse injury, similar to the injury that occurs on the battlefield [6].

Identifying mild to moderate TBI has always been challenging for researchers because it mainly depends on neurological severity score and Glasgow coma score [7]. The level of biochemical markers like superoxide dismutase, catalase, lipid peroxidase, glutathione, and histology of brain tissues also help determine the severity after TBI [8]. TBI is also detected by CT scan, PET, and MRI. But still, like always, there is a scope for improvement, which leads to inventions and possible identification measures, such as determining the reduced level of rnRNA in the brain during postmortem analysis [9, 10].

Various medicinal plants also show neuroprotective properties because they contain many active constituents. And these active constituents can reduce neuronal injury through various mechanisms, such as elevating antioxidant enzymes' levels of superoxide dismutase and catalase and decreasing the level of pro-inflammatory cytokines and neuro-inflammation, as shown in tables. The list of reported medicinal plants from 1976 to 2021 have been searched from various databases and are effective against TBI is mentioned in Table **1**, constituting data name of the plant, extract/or active constituents. These experimental models were used to induce injury and mechanism of action. Although these plants demonstrate very potent therapeutic activity in preclinical and clinical studies but also show some interaction with other drugs. More study is still required to completely know these agents' effects on TBI treatment.

TBI produces primary and then secondary injury. Primary injury cannot be reversed, so we try to reduce the secondary injury cascade with various synthetic drugs [11]. In TBI, no single synthetic drug reduces the progression of secondary injury; therefore, symptomatic treatment is given. For example, seizures occur due to glutamate toxicity. In that case, sodium valproate is given, and anti-fibrinolytic drugs like tranexamic acid are used if bleeding occurs at the time of injury. TBI causes injury and sometimes affects various parts of the brain, causing other problems to arise; therefore, a treatment plan should be given accordingly [12]. Synthetic drugs also produce adverse effects and drug interactions. In a nutshell, no specific drug is present that treats the major pathological events of TBI, like reducing ICP and NMDA receptor antagonist properties, increasing blood and oxygen level in brain tissue after injury, and reducing inflammation. The present treatment is ineffective in treating TBI due to the above mentioned limitations. More research is also required to provide better pharmacological intervention for TBI patients [13].

Traumatic brain injury is a complex neurological disorder of the brain caused by rapid movement within the skull, leading to damage with symptoms of mild to moderate TBI, including nausea, headaches, amnesia, dizziness, *etc*. This symptom resolves in days to weeks depending on the severity, whether mild, moderate, or severe. It is a leading cause of death in young adults and children. About 69 million people sustain TBI each year, in which road traffic collision is more common among individuals. Preventive steps and management of this disorder are still challenging [14]. In TBI, the damage is induced by direct or reversible secondary mechanisms. Due to primary insult, sudden mechanical damage of the brain tissue occurs and which causes delayed pathogenic events which collectively mediate widespread neuro-degeneration. The primary injury is responsible for initiating the secondary injury process that disperses *via* multiple molecular mechanisms in the pathogenesis of traumatic brain injury. The secondary injury occurs a few minutes after the primary injury, as shown in Fig. (**1**). These events lead to both neurodegeneration as well as functional recovery after TBI and are divided into four categories:

1) Primary injury, which destroys brain tissue.

2) Secondary injury occurs after primary injury and causes further brain damage.

3) Inflammatory reactions contribute to neurodegeneration.

4) Repair and regeneration that can lead to neuronal repair and regenerate them to some degree traumatic brain injury [15].

## 3 CLASSIFICATION OF TRAUMATIC BRAIN INJURY BASED ON MECHANISM

### 3.1 Focal Brain Injury

It occurs when any penetrating or sharp object strikes the brain resulting in a lesion, bruise, and intracranial bleeding.

### 3.2 Diffuse Brain Injury

It occurs due to acceleration/deceleration that results in diffuse axonal damage and swelling of the brain [16-18].

## 4 CLASSIFICATION OF TBI BASED ON SEVERITY

TBI severity is typically categorized using the Glasgow coma scale (GCS) score.

### 4.1 Mild Injury (13-15)

In mild TBI, unconsciousness occurs for half an hour or less, and post-traumatic amnesia persists not more than 24 hours. Transient confusion, disorientation, and cognitive dysfunction were also observed in the mild head injury.

### 4.2 Moderate Injury (9-12)

In moderate TBI, unconsciousness occurs for around half an hour to 24 hours, and post-traumatic amnesia persists from one day to 7 days.

### 4.3 Severe Injury (3-8)

In severe TBI, loss of consciousness occurs for more than 24 hours, and post-traumatic amnesia persists for more than 7 days [19, 20].

## 5 GENERAL PATHOPHYSIOLOGY OF TBI

After TBI, the initial stage in its pathophysiology is identified by direct tissue destruction that leads to abnormal regulation of CBF and metabolism [21]. Due to impaired CBF, there is a decrease in blood and oxygen in the brain, which ultimately leads to an ischemia-like situation. Due to decreased oxygen, anaerobic glycolysis occurs, resulting in lactic acid accumulation, increasing membrane permeability, and edema. The anaerobic process of metabolism does not provide sufficient energy to cells. Hence, cells uptake energy from ATP, which is already stored, resulting in ATP energy stores depleting and the stoppage of energy-dependent ionic pumps occur. The next step in pathophysiological events of TBI is identified by terminal depolarization of the membrane along with the massive discharge of excitatory neurotransmitters like aspartate & glutamate, which activates NMDA and AMPA receptors along with these voltage-dependent calcium channels. Successive sodium and calcium influx lead to auto-digesting intracellular processes. An excessive amount of calcium activates the lipid peroxidases, proteases, and phospholipases, ultimately elevating the amount of free fatty acids and free radicals intracellularly. Collectively, all these changes destroy the vascular and cellular structure, and finally, the process of apoptosis and necrosis occurs [22].

## 6 SPECIFIC PATHOPHYSIOLOGY OF TBI

This describes primary and secondary injury.

### 6.1 Primary Injury

The primary injury is demonstrated by focal injury, further represented by intracranial hematoma, laceration, and skull fractures, and diffuse injury, which causes mechanical harm to the brain. TBI resulting from a primary injury is determined to occur in a short span, *i.e*., 100 milliseconds [23]. These processes promote the shearing of blood vessels and axons generated by the injury. Intracerebral bleeding results in hemorrhage and breakdown of brain blood vessels, resulting in mass lesions, as shown in Fig. (**2**).

#### 6.1.1 Types of Primary Injuries

##### 6.1.1.1 Intracranial Hematoma

Intracranial bleeding is a common cause of damage and destruction after a primary injury. Various types of intracranial hemorrhage can occur after TBI and containing the following:

Intracranial hemorrhage means bleeding within the brain; it mainly occurs in the cerebral parenchyma inferior to lacerations, with destruction superior in deeper cerebral vessels happening through a broad cortical contusion [24].

An epidural hematoma occurs in bleeding between the dura and skull area, which mainly results from injury loading into the skull area through related laceration of the dural veins and arteries, usually *via* fractured bones and rarely by diploic veins within the skull’s marrow. One common reason for epidural hematoma is the tearing of the middle meningeal artery because of the fractured temporal [25]. It is arterial bleeding through a speedy increase in pressure [26]. When hematoma results from a laceration of the artery, immediate neurological damage results from blood collection and is dangerous in acute stages after TBI [27].

#### 6.1.2 Skull Fractures

In young adults, 10 to 30 percent of head injuries result in skull fractures. Fractured are defined as depressed and non-depressed, based on whether or not the inward displacement of fragments occurs. One bone fragment is a simple fracture; when two or more bone fragments arise, it is called a compound fracture.

In most cases, TBI results in skull fractures, which may correlate with cranial nerve damage, hemorrhage, and increased brain injury. Nearly 4 percent of all brain injuries cause fractures to the base of the skull [28]. Almost 90 percent of fractures are related to closed head injury, while the rest cause penetrating trauma [29].

Skull fractures, which mainly occur at the skull base, are responsible for injury to the cranial nerves. Facial nerves, a part of cranial nerves, are usually damaged after traumatic brain injury. Skull fractures may be responsible for damaging the brain's membrane and resulting in cerebrospinal fluid leakage (CFS). Due to intracranial leakage of cerebrospinal fluid, there is a generation of subdural hygroma. Extracranial cerebrospinal fluid leaks from the nose and ears, allowing bacteria and air to enter the skull and resulting in brain infection.

### 6.2 Secondary Injury

#### 6.2.1 Edema

After TBI, the frequent formation of edema occurs. Brain edema formation is related to structural deformity in the brain or water and osmotic imbalance due to primary and secondary injury [30]. Brain edema is of two types.

1) Vasogenic edema (interstitial)

2) Cytotoxic edema (intracellular)

Both types of edema occur after TBI, leading to further secondary impairment. After 24-48 hours of traumatic brain injury, the edema is at its worst. Cytotoxic edema is more common than vasogenic edema in TBI victims; the consequences of this edema are an increase in intracranial pressure and secondary ischaemic actions [31].

Vasogenic edema of the brain is induced by mechanical and autodigestive destruction or functional distraction of the brain vessel’s endothelial cell layer (ECL), an integral BBB structure. Decomposition of the cerebral vascular endothelial wall enables the unregulated movement of ions and proteins from intravascular to extracellular compartments of the brain, ensuring water collection [32].

This pathological process elevates fluid volume in extracellular space [33]. Cytotoxic edema of the brain is recognised by intracellular water collection into neurons, microglia, and astrocytes. Cytotoxic edema results from changes in cellular osmolality when the cell fails to maintain its ionic ingredients. This pathological process occurs due to a rise in permeability of the cell membrane for ions, failure of ionic pumps due to a decrease in energy, and reabsorption of osmotically active solutes by the cell damage mechanism due to excessive intracellular calcium overload [34]. These pathological changes trigger catabolic processes, including a breakdown of BBB, fastening Na^+^K^+^/ATPase action, and after that, metabolic requirement producing an intense loop of flow metabolism uncoupling to the cell [35]. The pathophysiological event of secondary brain injury is shown in Fig. (**3**).

#### 6.2.2 Oxidative Stress

It is defined as disequilibrium between generations of free radicals as well as the capability of the body to decontaminate their detrimental effects by neutralizing them through antioxidants [36]. Oxidative stress includes producing reactive oxygen species (ROS), which produces oxygen free radicals and other species such as superoxide, peroxynitrite, nitric oxide, and hydrogen peroxide in response to TBI [37]. The two major events in the pathophysiology of TBI, *i.e*., excitotoxicity and decrease in endogenous antioxidant enzymes such as catalase, superoxide dismutase, and glutathione peroxides, cause massive production of reactive oxygen species. All these events are responsible for protein oxidation, peroxidation of cellular and vascular structure, DNA cleavage, and the blocking of the mitochondrial electron transport chain (ETC) [38]. These pathological cascades are sufficient to contribute to instant cell death; oxidative stress is also responsible for neuroinflammatory processes and early or late program of cell death, *i.e*., apoptosis [39]. Oxidative stress is not limited to TBI only because it damages the structure and function of neurons [40]. It also affects various proteins and cell signaling pathways, which causes neural damage, leading to other neurodegenerative disorders like Alzheimer’s, and Parkinson’s [41].

#### 6.2.3 Cerebral Oxygenation

Traumatic brain injury is identified by a disturbance between the delivery of oxygen to the cerebral and consumption of oxygen to the cerebral [42]. In various vascular and hemodynamic processes, as mentioned earlier, the ultimate output is brain tissue hypoxia [43]. If any patient suffers from TBI and has brain tissue oxygen pressure less than 10-15mm Hg Pto_2,_ then the infarction of neuronal tissue occurs [44]. These events, like incidence, duration, and extent of tissue hypoxia, correlate with poor outcomes [45]. Sometimes deprivation of oxygen in the brain after secondary damage to the brain even occurs in a normal level of (ICP) intracranial pressure and cerebral perfusion pressure. Various clinical protocols integrate the parameter of brain tissue oxygen pressure into executive algorithms as guided by intracranial pressure or cerebral perfusion pressure. It further gives very useful information about the connection between cerebral oxygen delivery and demand and establishes better outcomes from traumatic brain injury. These results are seen when individualizing treatment depends on the oxygenation of vital brain tissues [46].

#### 6.2.4 Cerebral Vasospasm

After TBI, cerebral vasospasm is a valid secondary injury that detects actual patient outcomes [47]. Vasospasm produces in about 30 percent of TBI victims and results in severe brain injury and hypo perfusion in approximately 50 percent of patients who undergo vasospasm [48]. The mechanism for producing vasospasm includes long-term depolarization of vascular smooth muscles due to a decline in K^+^ channel movement, release of the endothelin due to a reduction in the availability of nitric oxide, and due to reduction of cyclic GMP in vascular smooth muscles [49]. These potentiating of prostaglandin-induced vasoconstriction and generation of free radicals are also responsible for vasospasm production. Thus, a decrease in death and disability is better attained by reducing the process of secondary insult, resulting in potential brain ischemia after vasospasm [50].

#### 6.2.5 Mitochondrial Dysfunction

Malfunctioning of mitochondria occurs due to the generation of free radicals, which further causes apoptosis (programmed cell death) after brain injury [51]. In TBI patients, there is a decrease in cellular energy production of cells because TBI causes ischemia in the brain so that less oxygen reaches; due to decreased oxygen, anaerobic metabolism occurs and produces lactate. Lactate does not provide sufficient energy to cells, so decreased energy of cells reduces, which causes impairment in neurological function [52]. Excessive release of glutamate induces neurotoxicity, which further causes mitochondrial damage in neuronal cells [53]. The mechanism by which mitochondrial dysfunction occurs after TBI is the massive discharge of glutamate, which cause activation of the NMDA receptor and results in the release of excessive intracellular calcium, which causes the overloading of calcium in mitochondria [54]. This extra calcium increases energy failure, and these processes cause harmful effects on mitochondria [55]. The calcium overload changes mitochondrial function and produces reactive oxygen species (ROS), reactive nitrogen species (RNS), and other free radicals. The resulting free radicals generally hit the calcium-overloaded mitochondrial neuronal cells, causing alteration in protein and development of mitochondrial membrane lipid peroxidase. The consequences of all these processes are a decrease in the calcium buffering ability of mitochondria, mitochondrial respiration, oxidative phosphorylation, and transport of ions due to the irreversible loss of mitochondrial processes [56]. Although the development of mitochondrial permeability transition pores is one of the important devastating sequences of calcium load into the inner membrane of mitochondria, dumping the matrix calcium pool back into the cytoplasm [57]. The mitochondrial permeability transition pore causes a rise in the inner mitochondrial membrane permeability (MMP), allowing solutes having a molecular mass of fewer than 1500 Daltons to cross smoothly through the inner membrane of mitochondria. These mitochondrial collapses decrease the cytoplasmic pool of adenosine triphosphate (ATP), which enhances energy failure, amplifies the elevation in cytosolic calcium, and delays calcium irregulation. Moreover, glutamate neurotoxicity, the process of mitochondrial malfunctioning, represents the mechanism of cell death, necrosis *versus* apoptosis, and neuronal cell death. Generation of mPTP after injury also produces oxidative stress components, which further promote the process of apoptosis by modifying mitochondrial cytochrome c release [58].

#### 6.2.6 Nitrite Level

Nitric oxide is the smallest biological molecule of mammals and is unique in nature. It is a gaseous chemical messenger with various physiological functions like intracellular signaling, cerebral regulation, intraneuronal communication, and neurotransmission release. Nitric oxide (NO) is synthesized from the amino acid l-arginine with the help of an enzyme, *i.e*., nitric oxide synthase (NOS).

There are three isoforms of NOS is present

• Endothelial nitric oxide synthase (eNOS)

• Neuronal nitric oxide synthase (nNOS)

• Inducible nitric oxide synthase (iNOS)

eNOS and nNOS are calcium-dependent; where iNOS is calcium-independent, its regulation is upgraded under inflammatory conditions. It is challenging to measure nitric oxide directly in the body because NO has a short half-life; it rapidly decomposes into nitrite and nitrate as a stable end product. So, NO is quantifiable by estimating NOx (Nitrite + nitrate) in biological samples. Estimating NOx is the indirect method for the measurement of NO in biological fluid; when a brain injury occurs, there is a rapid increase in the level of nitric oxide, which causes depolarization due to the opening of ion channel opens and increases intracellular calcium ions [59], NO plays a very important role in cerebral regulation. A low level of nitric oxide causes vasoconstriction and ultimately reduces cerebral blood flow, whereas an increased level of NO causes cytotoxicity in the brain because NO is metabolized to peroxynitrite, and this peroxynitrite further increases oxidative stress and ultimately causes cell damage [60].

#### 6.2.7 Inflammation

Traumatic brain injury induces a multifaceted adjustment of inflammatory as well as immunological tissue responses. The injuries, *i.e*., primary and secondary, activate the liberation of mediators of cells like prostaglandin, pro-inflammatory cytokines, free radicals, and other components. Along with these, chemokines and adhesive molecules stimulate mobilizing immune and glial cells in a similar and symbiotic pattern [61]. For instance, activated polymorpho-nuclear leucocytes remain defective, and adhesion molecules mediate the complete endothelial cell layers. These cells infiltrate damaged tissues along with macrophages and T-cell lymphocytes [62]. These tissues' infiltration occurs by upregulating cellular binding molecules such as VCAM-1 (vascular adhesion molecules), ICAM-1 (intracellular adhesion molecules), and P-selectin. In response to these inflammatory responses, the damaged tissues (depending upon ‘spreading depression’) will be removed within a few hours, days, and weeks; astrocytes produce microfilaments and neutropines to synthesize scar tissues. Within a few hours of brain injury, pro-inflammatory enzymes like TNF-α, IL-β, and IL-6 are upregulated. The elevation in tissue destruction is directly related to liberation of neurotoxic mediators and indirectly correlated with a discharge of cytokines and nitric oxide. Further discharge of vasoconstrictors like prostaglandins and leukotrienes, damaging of microvasculature by binding of platelets and leucocytes, the lesions on BBB, and development of edema further decrease tissue perfusion and result in more worsening secondary damage to the brain [63]. After TBI, the inflammation affects not only the central nervous system but also the peripheral system. The cells of the CNS, like, astrocytes and microglia, are also responsible for producing various other chemicals which cause inflammation in the brain and change its morphology [64]. The chemicals released after injury also change the brain cell microenvironment, and ultimately cell death occurs [65]. After moderate to severe injury, which stays for months to years, it also affects the quality of life and leads to other diseases like epilepsy, dementia, and depression [66]. In a conducted study, the cross of HDAC3^LoxP^ mice with CX3CR1^CreER^ mice for the removal of HDAC3 *in vivo* conditions. Microglia-specific HDAC3 knockout was induced in CX3CR1^CreER^: HDAC3^LoxP^ mice with 5 days of tamoxifen treatment followed by a 30-day development interval. HDAC3 miKO reduces 26% of microglia, but apparently, no change in the homeostatic brain was found from the perspective of inflammation. In HDAC3 miKO mice, signal conduction was enhanced by white matter [67].

#### 6.2.8 Necrosis versus Apoptosis

Apoptosis and necrosis are the types of cell death that occur after traumatic brain injury. In apoptosis, programmed cell death occurs, while in necrosis, cell death occurs due to external injury or tissue damage. Generally, after TBI, tissue destruction occurs due to ischemia or hypoxia along with this massive discharge of excitatory neurotransmitters like glutamate and metabolic failure, which is also responsible for necrosis [68]. Latterly, biological membrane autolysis occurs due to phospholipases, lipid peroxidases, and proteases. The resulting debris of cells is identified as an antigen and will be eliminated by the inflammatory pathways, leaving scar tissues behind. On the other hand, neurons that undergo apoptosis remain morphologically undamaged during the post-traumatic stage with the optimum production of ATP, which gives potential to the physiological membrane. So, after the primary injury, the process of apoptosis (programmed cell death) becomes evident in hours or days. Translocation of the phosphatidyl serine involves discrete but successive membrane damage together with the lysis of the nuclear membrane, DNA fragmentation, and chromatin condensation [69]. While an excess of intracellular calcium, an excitatory neurotransmitter, and free radicals can also be responsible for apoptosis because *in vitro* experiments demonstrated that neuronal cells can experience apoptosis through multiple pathways [70]. After the process of apoptosis, the resultant debris, like apoptotic contents, is eliminated from the shrinking cell by the exocytotic pathway. A disturbance between the anti and pro-apoptotic proteins is also responsible for apoptosis. Successive activation and inactivation of caspases that demonstrate particular proteases of IL-converting enzyme group has been characterized as the most consequential mediators of apoptosis. The clinical importance of programmed cell death (apoptosis) is linked to the delayed onset of cellular degradation, potentially providing a more target for therapeutic (antiapoptotic) intervention [71].

#### 6.2.9 Cerebral Metabolic Dysfunction

Cerebral metabolism is indicated by the intake of cerebral oxygen and glucose. In contrast, cerebral energy is indicated by concentrations of ATP and phosphocreatine in the tissue or indirectly by the lactate/pyruvate ratio. Both these variables are reduced after TBI and may remain with important structural and temporal heterogeneity [72]. Primary insult is the major reason for metabolic failure. Poor outcomes are seen in patients with low metabolic rates. The decrease of post-traumatic cerebral metabolism involves the primary (immediate) injury, which results in mitochondrial dysfunction. This is also followed by a decreased supply of the nicotinic coenzyme pool, reduced respiratory levels, intramitochondrial calcium overload, and ATP production [73]. However, hyperoxia is used for correcting metabolic failure but produces inconsistent outcomes. The decline in cerebral metabolic demand may or may not be linked to a decline in cerebral blood flow [74]. The latter represents the uncoupling of cerebral blood flow and metabolism, presumably due to the increased availability of adenosine. In a few cases, a distinct pathophysiological cascade, such as glucose hypermetabolism, may occur [75]. This is powered by transient but excessive ionic transmembrane fluxes with successive neuroexcitation, but these are not sufficiently met by a rise in cerebral blood flow. The secondary ischaemic insult development is assisted by the uncoupling of cerebral blood flow (CBF) metabolism [76].

#### 6.2.10 Cerebrovascular Autoregulation and Co_2_– reactivity

Maintenance of cerebral perfusion pressure and intracranial pressure can be carried out by carbon-dioxide reactivity and cerebrovascular autoregulation [77]. Both variables are crucial in providing sufficient cerebral blood flow [78]. The secondary injury disrupts these two regulating processes [79]. C cerebral autoregulation maintains cerebral blood flow even at distinct cerebral perfusion pressure. The difference between mean arterial pressure (AP) and intracranial pressure (ICP) is known as cerebral perfusion pressure (CPP) [80, 81]. This process is known as cerebral blood flow (CBF) autoregulation. This process is disturbed by many victims of TBI [82]. The generation of defective cerebrovascular autoregulation takes place after TBI. It may occur after a few hours of injury and could be persisted even with mild, moderate, or severe damage [83]. Autoregulatory vasoconstrictions are more resistant than vasodilation, representing that TBI victims are more prone to harm from low rather than high cerebral perfusion pressure [84]. For patients with severe head injuries and poor results, carbon dioxide reactivity is an imbalance in the initial stage after TBI [85]. On the other hand, carbon dioxide reactivity remains the same or even increases in some patients offering this physiological principle as a target for intracranial pressure management in hyperaemic states [86].

#### 6.2.11 Glucose Metabolism

After TBI, mechanical tissue destruction occurs, which causes decreased oxygen to brain cells; due to the absence of oxygen, anaerobic metabolism causes accumulation of lactic acid, reduced glucose utilization, reduction in ATP and action of ATPase pump, increased excitotoxicity as well as intracellular calcium, resulting in cell death [87]. Various experimental studies have shown that TBI causes a considerable increase in glucose utilization following the early half an hour after injury. After that, a decrease in glucose level is maintained in a low state for about 5 to 10 days [88, 89]. Decrease cerebral glucose metabolism was observed in both mild and severe TBI victims [90]. After TBI, ischemia occurs, which causes an increase in lactate level; it may produce neuronal malfunctioning because of acidosis, interruption of BBB, edema, and membrane damage [91, 92]. In addition, some manifestations reveal that lactate accumulation after TBI may make the neurons more vulnerable to secondary ischaemic insult [93].

Traumatic brain injury is one of the major reasons for morbidity and mortality [94]. It is a major public health concern worldwide and is expected to overtake other diseases by 2020 as a major cause of death and disability (WHO, 2002). The pathophysiological cascade of TBI is very complex. So, to treat these pathological symptoms, various synthetic drugs are used. These synthetic drugs do not show satisfactory therapeutic action but also have adverse effects.

Recently, various conventional TBI supplements and herbal medicine therapies were created. They involve crude extracts and isolated compounds, which have demonstrated neuroprotective effects. Reported studies from 1976 to 2021 have been searched from various databases on TBI herbal medication, which were mentioned in Table **1**, constituting data name of plant, extract/or active constituents, experimental models used to induce injury, and mechanism of action.

The inclusion and exclusion criteria of selected plants for the review were based on the therapeutic potential of plants in TBI. Those plants or active constituents acted *via* multiple mechanisms. They demonstrated a significant reduction in neuronal injury parameters, such as decreasing lipid peroxidase, IL-1, IL-6, nuclear factor kappa beta, TNF-alpha, increased catalase, and superoxide dismutase activities were taken.

Various phytoconstituents were also studied in treating TBI and showed a very potent therapeutic effect in preclinical studies. A list of phytoconstituents used in treating TBI is mentioned in Table **2**, along with their dose, experimental model, and mechanisms of action.

Although clear mechanisms for neurodegenerative disorders are still unclear, protein aggregation is strongly considered for the emergence and development. The presence of molecular development in abundance capacities has therapeutic potential to disturb aggregates and resist the misfolding of protein. But in neurodegenerative proteotoxic stress conditions, the cells do not or poorly adapt to maintaining the equilibrium of protein folding.

Peroxynitriles and hydroxyl radicals can absorb a hydrogen ion from fatty acids. The original radical gets quenched to form a lipid peroxyl radical that interacts with neighboring fatty acids in a self-propagating motion, resulting in lipid peroxidation of cellular membranes. By lipid peroxidation of the cellular membrane, 4-hydroxynonenal (HNE) gets produced, contributing to neurodegenerative disorders.

Plasma membranes' redox system controls the levels of oxidation stress. For modulation and controlling multiple metabolic signaling and transcriptional processes, protein thiols play a major mediator in redox sensing and regulation of cellular redox state.

Oxidizable diphenols are inducers of the Keap1/ NrF2/ ARE pathway and repress for NrF2. Oxidizablediphenols react with cysteine residues of Kep1 to repress the NrF2, resulting in NrF2 stability and cytoprotective genes.

In autosomal recessive early onset of Parkinsonism disorders, there is a loss of function mediators in PARK7, which encodes for DJ-1—impaired p62 expression results in a decrease in NrF2 activity, which results in neurodegenerative disorders.

Polyphenols such as Tert-butylhydroxyquinone, sulforaphane, Dimethyl fumarate, Diallyl trisulfide, celastrol, Curcumin, and ferulic acid have shown good therapeutic potential for the treatment of neurodegenerative disorders. Tert-butylhydroquinone has oxidation-reduction lability, which is essential for inducing the Keapl/ NrF2/ RE ability. Sulforaphane can induct NQ01 and inhibit the upregulation of inducible NO synthase and COX. Celastrol induces NQ01 and inhibits NFkB. It also reduces inducing the expression of class 2 HMC molecules by microglia. Curcumin has therapeutic potential for treating astrocytes by inducing HO-1, NQ01, and GST and protects against the damaging effects of glucose oxidase-mediated toxicity. Curcumin reduces amyloid levels and plaque burden in aged Tg2576 mice with advanced amyloid accumulation. Curcumin also down-regulates inflammatory transcription factors, enzymes, and cytokines. Curcumin also interacts with the vitagene system, where curcumin increases the expression of HO-1 in human cardiac myoblast, endothelial cells, monocytes, and hepatocytes [95]. Curcumin also has potent antioxidant properties; this property ameliorates TBI and induces oxidative stress. Combating oxidative stress increases the level of BDNF and cAMP and prevents the brain from further disease progression [96]. According to the literature, curcumin acts through various pathways in treating TBI, so it is very beneficial in treating TBI [97].

Other natural compounds like terpenoids also have a neuroprotective effect like monoterpenoids, *e.g*., thymol, α-terpineol, limonene, menthol, and carvone. Thymol is a dietary monoterpenoid, which is beneficial in treating various neurological disorders like Parkinson’s, epilepsy, and brain injury. Thymol shows potent pharmacological properties like anti-inflammatory, antioxidant, and analgesic. One major therapeutic effect is that it decreases infarction and increases blood perfusion after injury; due to these properties, it also shows neuroprotection [98].

As we discussed above, herbs that show the neuroprotective effect of another herbal plant, *Astragalus membranaceus,* which contain active constituent like Astragaloside IV in its roots, also have potent neuronal protective action. Astragaloside IV has antioxidant, anti-inflammatory as well as antiapoptotic properties. Due to these properties, it is beneficial in various neuronal disorders like Parkinson’s, Alzheimer’s, and brain injury [99].

## CONCLUSION

Researchers are working vigorously to understand the pathology of TBI and trying to discover and invent new drug molecules for treating TBI. Many mechanisms and theories have been reported by various scientists. But till now complete pathological mechanism has not been discovered. But some drugs have been marketed for treating TBI, but they are insufficient. Numerous reported studies have supported the potential of phytoconstituents of various herbal species against TBI. Therefore, phytopharmaceuticals can be potential agents for the treatment of TBI.

## Figures and Tables

**Fig. (1):**
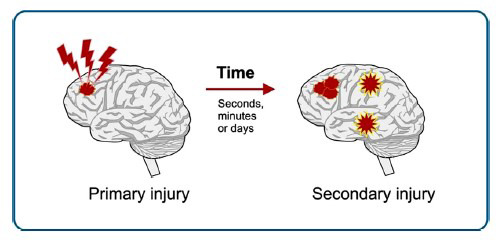
Effect of time on TBI.

**Fig. (2):**
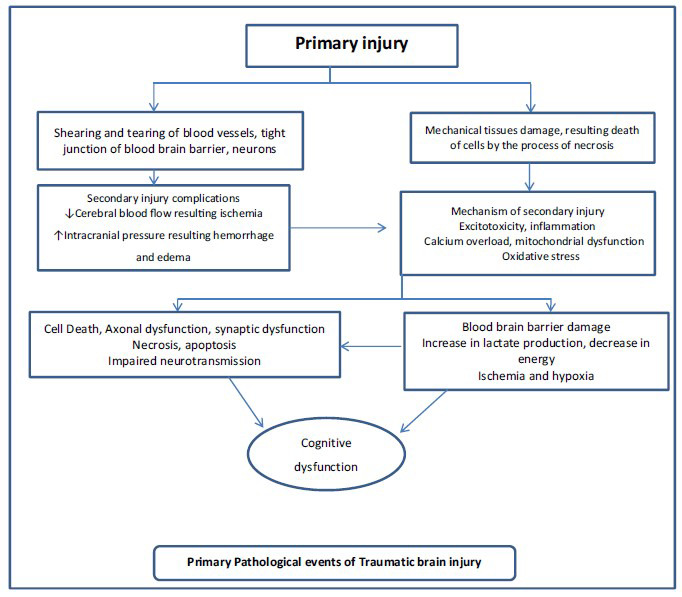
Primary pathological events of traumatic brain injury.

**Fig. (3):**
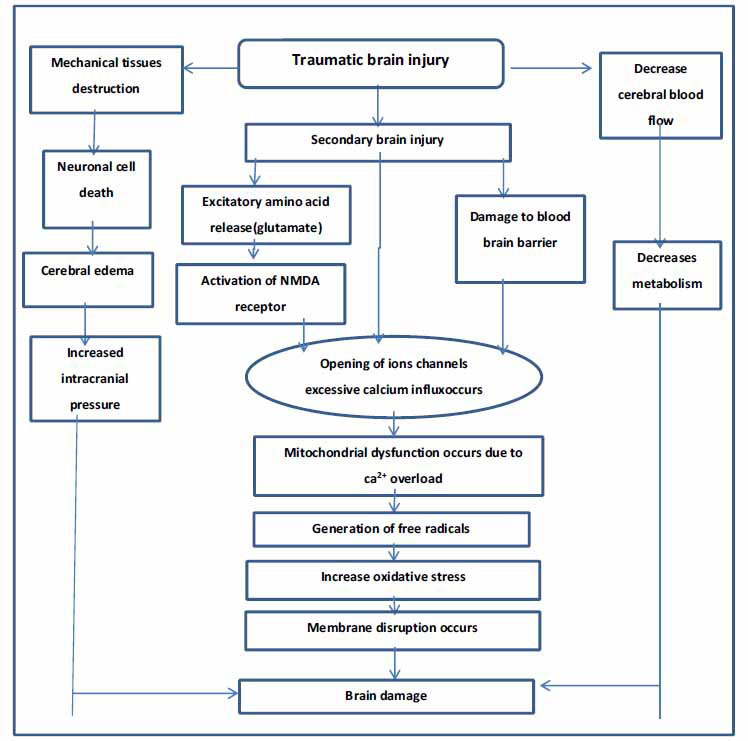
Pathophysiological events of secondary brain injury.

**Table 1:** List of medicinal plants which can effective against TBI.

**S. No.**	**Medicinal Plants**	**Extract/ Active Constituent**	**Dose**	**Experimental Model**	**Mechanism to Treat TBI**	**References**
1	*Allivum sativum*	Allicin	1, 10 and 50 mg/kg	CCIM	• Increased the level of antioxidant enzymes such as SOD, CAT and reduced MDA level. • Reduced water content, TNF, IL-6 and also inhibit activation of caspase-3.	[100, 101]
2	*Actaearacemosa*	Formononetin	10 and 30 mg/kg	WDM	• It improved NSS. • Reduced IL-6 and TNFα. • Decreased brain oedema and neurological function.	[102]
3	*Aframomummelegueta* seeds	Aqueous ethanolic extract	10, 100, 250, 500 and 1000 mg/kg	FPM	• It decreased microglial activation and neuronal injury.	[103]
4	*Artemisia annua*	Atesunate	30 mg/kg	CCIM	• It decreased inflammation and tissue damaged as well as IL, TNFα, iNOS and BDNF.	[104]
5	*Apocynumcannabinum*	Apocynin	5 mg/kg, i.p	WDM	• It decreased brain oedema. • Improved learning and memory ability by MWM test. • It also decreased NOX.	[105]
6	*Carthamustinctorius*	HSYA	10 and 30 mg/kg	CCIM	• It elevates the SOD, CAT, GSH level and reduced MDA and Nitrite.	[106]
7	*Cinnamomumzeylanicum*	Polyphenol E	10 mg/kg	CI	• It decreased oedema and infarct formation and also suppress the growth of cytokines such IL, TNFα *etc*.	[107]
8	*Cnidiummonnieri*	Osthole	10, 20 and 40 mg/kg	WDM	• Improved neurological deficit. • Elevate the level of SOD, GSH and decrease MDA. • Decreased cerebral oedema and neuronal loss.	[108]
9	*Crocus sativus*	Crocin	20 mg/kg	CCIM	• It decreased brain oedema and also improved NSS. • It cause reduction in cell apoptosis, microglia activation and also decreased release of IL-1β and TNFα.	[109]
10	*Coffee arabica*	Caffeine	25 mg/kg, i.p	FPM	• Improved NSS score. • Decreased in mortality.	[110]
11	*Coffee arabica*	Caffeine	0.25 g/L in the subject’s drinking water	FPM	• Improved NSS score. • It ameliorates oedema, decrease apoptosis and inflammation.	[111]
12	*Curcuma longa*	Curcumin	50, 100 and 200 mg/kg	WDM	• Improved NSS. • Decreased neuronal as well as apoptotic process of cell death and also decreases the microglial activation.	[112]
13	*Curcuma zedoaria*	β-elemene	100 mg/kg	WDM	• Ameliorated NSS. • It also decrease the level of inflammatory mediators and cytokines like IL-6 and TNFα.	[113]
14	*Colchicum autumnale*	Colchicine	0.2 mg/kg, i.p	WDM	• It decreased the level of iNOS and also decreased ROS. • It also reduced the number of FJB cells.	[114]
15	*Da Chuanxiong Formula (DCFX)*	Aqueous extract	520.6 and 2603.0 mg/kg	CCIM	• It decreased BBB permeability, microglial and astrocytes activation as well as brain oedema.	[115]
16	*Dracaena cochinchinenesis*	Aqueous extract	40 and 80 mg/kg	WDM	• Elevates the level of SOD. • Decreased the serum level of MDA, TNFα and IL-1β.	[116]
17	*Drynaria fortune*	Aqueous extract	20 mg/kg	CCIM	• It improved NSS score. • It blocked the activation of microglia and astrocytes. • It also decreased brain lesion volume and IL -6.	[117]
18	*Erigeron brevicapus*	Breviscapine	75 µg	CCIM	• NSS improved. • Decrease the IL-6 in the injured cortex.	[118]
19	*Gastrodiaelata*	Aqueous extract	505 and 1515 mg/kg	-	• It reduces the astrocytes and IL-6 & TNFα.	[119]
20	*Ginkgo biloba*	Extract	50 mg/kg, i.p	WDM	• It decreased the level of MDA.	[120]
21	*Ginkgo biloba*	Extract	5, 10 and 20 mg/kg	WDM	• It inhibits neuro-inflammation and oxidative stress. • It also reduced the release of inflammatory mediators like IL-6 and TNFα.	[121]
22	*Ginkgo biloba*	Extract	100 mg/kg, i.p	Pneumatic impactor	• Improved movement in MWM test. • It also decreased the activation of astrocytes and microglia.	[122]
23	*Malvasylvestris*	Extract	250 and 500 mg/kg	CCIM	• Increase SOD level. • Reduced neuronal loss, ROS level and IL-6, LPS, and GFAP Positive cells. • Increased cognitive function in MWM test.	[123]
24	*Nicotianatobacum*	Nicotine	0.125 mg/kg/hr	CCIM	• It decreased lesion size.	[124]
25	*Nigella sativa*	Thymoquinone	10 mg/kg	WDM	• It decreased LDH and plasma co-peptin level in the brain tissue.	[125]
26	*Panax ginseng*	Aqueous extract	50, 100 and 200 mg/kg	WDM	• It causes improvement in neurological deficit. • It elevates the SOD, GSH and CAT level. • Decrease in MDA, nitrite and IL & TNF-α level.	[126]
27	*Papaversomniferum*	Morphine	10 mg/kg, i.p	FPM	• It enhances the motor skill.	[127]
28	*Papaversomniferum*	Morphine	15 mg/kg, i.v	CCIM	• It do not show improvement in MWM test. • Do not improve NSS.	[128]
29	*Pinusmaritima*	Pycnogenol	100 mg/kg, i.p	CCIM	• It elevates antioxidant enzymes such as SOD, CATandGPx while decrease the MDA and other cytokines like interleukins and tumour necrosis factorα.	[129]
30	*Pleurotusostreatus*	Lovastatin	4 mg/kg, i.p	CCIM	• Lovastatin decreased the level of IL-6 and TNFα. • It improved neurological function.	[130]
31	*Polygonum cuspidate*	Emodin	10 mg/kg	WDM	• Decreased BBB permeability as well as brain oedema.	[131]
32	*Rheum tanguticum*	Aqueous extract	3, 6 and 12 mg/kg	CCIM	• Reduced BBB damage and oedema of brain. • Increase SOD, Cat, GSH and decrease MDA & GSSH.	[132, 133]
33	*Rosmarinusofficinalis*	Aqueous extract	40, 80 and 160 mg/ml	LFP	• It suppressed the neuronal degeneration, ROS generation and level of pro-inflammatory mediators. • It elevates the level of SOD, CAT and GPx.	[134]
34	*Salvia tomentosa*	Luteolin	20 mg/kg	CCIM	• Decrease in level of TNFα as well as IL-1β in blood and in brain tissue.	[135]
35	*Salvia miltiorrhiza*	SalB	25 mg/kg	CCIM	• Improved neurological function. • Reduced oedema and inflammation.	[136]
36	*Saturejakhuzistanica*	Essential oil	50, 100 and 200 mg/kg	WDM	• It decreased brain oedema and repaired the damaged BBB and also ameliorated the veterinary coma scale score. • It also reduced neuronal death and decrease BBB permeability. • It reduced level of TNFα, IL-6 and IL-1β.	[137]
37	*Scutellariabaicalensis*	Baicalein	30 mg/kg	CCIM	• It improved neurological functioning. • It also reduces expression and also release of pro-inflammatory mediators.	[138]
38	*Scutellariabaicalensis*	Wogonin	20, 40 and 80 mg/kg	CCIM	• It improved neurological functions. • It decreased brain oedema, pro-inflammatory cytokines like IL-6, TNFα and also reduced BBB permeability.	[139]
39	*Tripterygiumwilfordii hook*	Triptolide	0.125-1.0 mg/kg	CCIM	• It decreased oedema and neuroinflammation.	[140]
40	*Zanthoxylumnitidum*	Nitidine	2.5 mg/kg, i.p	Injury induce by inserted 21gm needle	• It decrease microglial activation.	[141]

**Table 2:** List of phytoconstituents which can effective against TBI.

**S. No.**	**Phytoconstituents**	**Dose**	**Experimental Model**	**Mechanism to Treat TBI**	**References**
1	Caffeic acid phenyethyl ester	10 µmol/kg	WDM	It decreased the level of MDA and increased the antioxidants level like SOD. It also reduced TUNEL staining in frontal cortex.	[142]
2	Ellagic acid	100 mg/kg	WDM	It decreased the permeability of BBB and level of cytokines like IL-6 and TNFα.	[143]
3	Formononetin	20 mg/kg	WDM	It decreased the level of MDA and increased the antioxidants level like SOD. It also reduces release of inflammatory cytokines such as IL-6, TNF-α.	[144]
4	Genistein	15 mg/kg, i.p	WDM	It reduced brain edema as well as opening of BBB. It also decreased intracranial pressure.	[145]
5	Gallic acid	100 mg/kg, orally	WDM	Improved NSS score. Decreased the level of pro-inflammatory cytokines.	[146]
6	Icariin	60 mg/kg, orally	CCIM	It reduced the inflammatory markers like IL-6, TNF-α, COX-2, IL-1β.	[147]
7	Luteolin	30 mg/kg, i.p	WDM	Luteolin reduces brain edema, neuronal degeneration, BBB disruption. It also reduces release of pro-inflammatory markers likes IL-1b and TNF-α.	[148]
8	Naringenin	100 mg/kg, i.p	WDM	Naringenin reduces endoplasmic reticulum occur stress related protein expression after TBI, it also reduces brain edema, oxidative stress, cell death.	[149]
9	Quercetin	30 mg/kg, i.p	WDM	Quercetin significantly decreases the oxidative stress and pro-inflammatory mediators such as IL-6 and TNFα. It also increased the level of SOD, CAT and GPx. It elevates the level of BDNF and improved activity in MWM test.	[150]
10	Quercetin	50 mg/kg, i.p	WDM	It attenuates cerebral edema, neuronal apoptosis in cortex, and also reduces TBI induces motor deficit.	[151]
11	Resveratrol	100 mg/kg, s.c	WDM	It decreases microglia activation It also reduced pro-inflammatory mediators such as IL-6 & 12.	[152]
12	Resveratrol	100 mg/kg	CCIM	It reduced injury volume and also protects neurons. It reduced the generation of ROS. It decrease edema and enhance motor and coordination behaviour. It also improved activity in MWM test.	[153]
13	Resveratrol	100 mg/kg, i.p	WDM	It decrease edema and enhance motor and coordination behaviour. It also improved activity in MWM test. It also reduced cytokines level such as IL-6 & 12, TNFα.	[154, 155]
14	Resveratrol	100 mg/kg, i.p	CCM	It decreased edema and oxidative stress.	[156]

## LIST OF ABBREVIATIONS

AD: Alzheimer's Disease
ATP: Adenosine Triphosphate
BBB: Blood-brain Barrier
BDNF: Brain Derived Neurotropic Factor
CBF: Cerebral Blood Flow
CCIM: Controlled Cortical Impact Model
DAI: Diffuse Axonal Injury
FPM: Fluid Percussion Model
GCS: Glasgow Comma Scale
HDAC: Histone Deacetylases
ICAM: Intracellular Adhesion Molecules
ICP: Intracranial Pressure
IL: Interleukin
miKO: Microglia-specific Knockout
NMDA: N methyl D Aspartate
PBBI: Penetrating Ballistic Like Brain Injury
ROS: Reactive Oxygen Species
TBI: Traumatic Brain Injury
TNF: Tumour Necrosis Factor
VCAM: Vascular Adhesion Molecules
VEGFA: Vascular Endothelial Growth Factor Activator
WDM: Weight Drop Model
